# Blockchain technology: development and prospects

**DOI:** 10.1093/nsr/nwy133

**Published:** 2018-11-13

**Authors:** Weijie Zhao

**Affiliations:** NSR news editor

## Abstract

It has been more than 10 years since Satoshi Nakamoto published his famous paper entitled ‘Bitcoin: a peer-to-peer electronic cash system’, which set the foundation of blockchain technology. Accompanied by the price volatility of bitcoins from 2017 to 2018, blockchain has been a hot word on the internet, and particularly hot in China. Blockchain offers a distributed and secure system for data storage and value transactions. Its applications are springing up in multiple fields.

The Chinese government is considering these trends with great caution. Initial coin offering has been banned in China since September 2017. By contrast, an official white paper on China's blockchain technology, which was released in May 2018, said that blockchain technology will be widely applied in the real economy of China within 3 years. In a recent panel discussion held by *National Science Review*, experts talked about related topics. Their opinions may provide a quick view of the future development of blockchain in China and abroad.

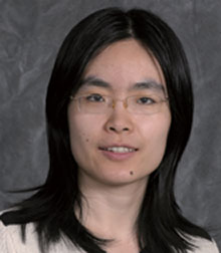

Jing Chen

Assistant Professor of Computer Science Department, Stony Brook University and Chief Scientist at Algorand LLC, USA

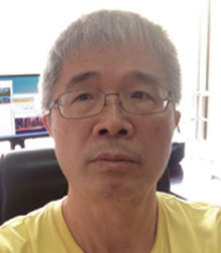

Xiaotie Deng

Professor of School of Electronics Engineering and Computer Science, Peking University, China

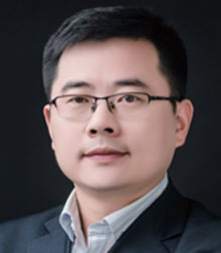

Guohua Gan

Vice President of Beijing Tai Cloud Technology Corp., China

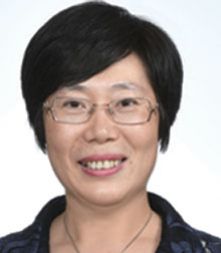

Xiaoyun Wang

Professor of Institute of Advanced Study, Tsinghua University, China

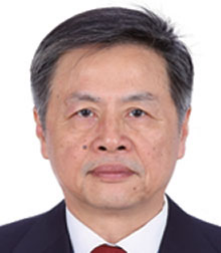

Zhiming Zheng

Professor of School of Mathematics and Systems Science, Beihang University, China

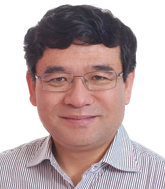

Lei Guo (Chair)

Professor of Academy of Mathematics and Systems Science, Chinese Academy of Sciences, China

## MULTIPLE APPLICATIONS OF BLOCKCHAIN

**Guo:** Blockchain has attracted wide attention from academia, industry, government and the public. So the first topic of our discussion is, what can blockchain do and why are so many people interested in it?

**Zheng:** I would like to talk about people's interest in this technology. We have observed the interconnection of information since the beginning of the internet era. And then with the ubiquity of smartphones, the interconnection of people became a reality. Now, fifth-generation mobile communications (5G) technologies and Internet of Things (IoT) technologies are fast developing, and we will head into an everything-connected era within several years.

After we have connected information, people and things, what will be the next step? It should be the connection and transaction of value. The basis of value connections is trust. So people are eager for a technology that could ensure trust and set up a low-cost passageway for value transaction. Blockchain offers a secure and tamper-proof tool for the storage of data and transaction of value; thus, it solves the trust problem at the mechanism level.

The establishment of a trust mechanism could also promote economic growth. The great economist Philip Fisher discussed

Blockchain can be applied to a certain application scenario provided the scenario has one of the following six properties: it needs multiparty interaction; it needs creditability; it needs disintermediation; it needs atomicity; it needs privacy; or its production relations need essential adjustment.

—Zhiming Zheng

the relationship between economic growth and the pricing of value. Milton Friedman, the promoter of monetarism, further explained Fisher's idea, and his explanation can be summarized as follows: gross domestic product (GDP) equals the product of money supply, which could be narrow money supply M1 or broad money supply M2, and the velocity of value flow. The establishment of a trust mechanism can speed up value flow and thus speed up the growth of GDP.

**Gan:** Blockchain technology can be used in many fields. Its current applications include transaction, payment, data storage and certification, track and trace, and smart contract. In my opinion, these applications can be classified into two categories: applications purely on the chain and applications that combine on-chain and off-chain issues.

Virtual currency is a typical purely on-chain application. The token is born and transacted completely on the chain. Track and trace is an example of a combination of online and offline business. Several months ago, there was a public issue over unqualified vaccines in China. Someone proposed that we could use blockchain technology to create traceable records of the full life cycle of a vaccine, from production to transport to usage. For this type of application, a critical point is to guarantee consistency between on-chain tagged items and off-chain real-world items.

**Zheng:** That is right. I think that blockchain can be applied to a certain application scenario provided the scenario has one of the following six properties: it needs multiparty interaction; it needs creditability; it needs disintermediation; it needs atomicity; it needs privacy; or its production relations need essential adjustment. There are diverse eligible scenarios, including waste management, supply chain management, government management, culture and entertainment, and intelligent manufacture, as well as public welfare. The application field of blockchain is far beyond the financial sector.

**Deng:** Among all these applications, I think the purely online applications, which are completely electronic and digitalized, will become a reality the easiest. Examples include applications in e-finance and electronic resource sharing, as well as internet-enabled services and games.

One possibility is the use of blockchain to develop solutions to achieve both social optimality and individual privacy. The internet has provided more efficient social services; however, an issue is how to preserve individual privacy and at what cost. There are cases in which internet services have improved the overall welfare of customers, but in certain circumstances, the technology has failed to meet common people's expectations. A challenge in the progress of internet services is whether we want Pareto improvement in everyone's welfare instead of mere total social welfare. However, this is often not possible, and a trade-off has to be made in which one is sacrificed for the other.

Google's motto ‘Don’t be evil’ declares the market maker's promise to society and individuals. However, could this be achieved not only by a company's goodwill but also be enforced by the mechanism design, combining the technologies of blockchain, big data and IoT to develop future automated human service systems?

**Chen:** Blockchain, when designed properly, allows consensus to be reached efficiently in a large-scale distributed system. Consensus is the basis of many applications closely related to people's daily life, from finance to data sharing. People need to agree on who owns what and who has done what to be able to continuously communicate and collaborate. Blockchain provides a novel way of achieving distributed consensus. That's why it is relevant to so many aspects of daily life.

Consensus is one pillar of a secure and healthy blockchain environment. Mechanism design is another pillar. Academia has proposed many well-designed and high-quality theoretical mechanisms. However, the mechanisms used in the real world are still traditional and simple. This is the result of the public's limited receptivity. It is difficult to apply a complex mechanism in a giant economic system. The public will not understand and accept the mechanism easily, and they will not trust the service and suggestions offered by the mechanism. So in the mechanism design of blockchain, being simple and being interpretable should be important principles. To meet these principles, we may have to compromise on its optimality.

## THE ALGORITHMS SUPPORTING BLOCKCHAIN

**Guo:** Blockchain technology is secure, tamper-proof and decentralized. All of the applications mentioned above are based on these properties. So what are the basic algorithms that support these properties?

**Wang:** Let me talk about the cryptographic technologies in blockchain. We use cryptographic technologies to guarantee the security of communication, and there are three major cryptographic technologies used in blockchain: encryption algorithm, digital signature technology and the hash function.

Encryption algorithm can ensure the confidentiality of information and guarantee that a message cannot be seen by others. Digital signature technology can ensure authenticity, data integrity and non-repudiation. The hash function can guarantee

There are three major cryptographic technologies used in blockchain: encryption algorithm, digital signature technology and the hash function.

—Xiaoyun Wang

that the data recorded on the blockchain cannot be tampered without detection. Moreover, the tamper-resistant property of the digital signature is also achieved with the help of the hash function. Tamper-resistance is a basic property of blockchain, so the hash function plays a central role in blockchain technology.

The hash function has many advantages as a cryptographic primitive. First, it is highly secure and also easy to use. The hash function does not need a key and can be easily used by everyone in many scenarios. Simultaneously, it possesses three major security properties: preimage resistance, second preimage resistance and collision resistance. It can provide high security without relying on a key.

Second, the hash function is typically modeled as a random oracle in the security proofing of cryptographic systems. Comparing with traditional provable cryptographic protocols, such as the zero-knowledge proof, the hash function can transform an interactive proof system into a non-interactive one where the prover needs to deliver a message only once. Hence, the communication complexity can be reduced significantly.

Third, the hash function can benefit the binding property. The binding property is a part of the commitment schemes, which proceed as follows: I send you a commitment, say, the hash value of a message; and then once you have received the corresponding message from me, you can check that whether the hash value matches the message. The binding technique is applied in transaction, payment, digital certification, track and trace, as well as smart contract to ensure the security of these systems, and it is very important for the application of blockchain technology.

**Chen:** There are also new algorithms that have appeared in recent years. In traditional blockchain technology, there is a so-called ‘trilemma’. People believe that security, performance and decentralization cannot be obtained simultaneously; we have to sacrifice one to achieve the other two. Bitcoin and Ethereum (ETH) both follow this rule. But now, some new technologies, such as Algorand's new consensus system, make it possible to achieve these three properties simultaneously.

Our new consensus system is no longer based on Nakamoto's proof of work mode, but on a specially designed Byzantine agreement and a proof of stake mode. For security, we can mathematically prove that the system is safe with no more than one-third malicious stake, regardless of what the adversary does. Safe means that the transaction information cannot be tampered with, the chain will not fork and a new block will be recognized as the final block as soon as it is created. For performance, as we no longer use the proof of work method, our chain does not rely on mining or the solving of the hash function. So the amount of calculation needed is quite low. Everyone with a laptop computer and basic internet connection can join the system as a full function node. And for decentralization, we did not compromise, as in other systems based on delegated proof of stake, which select a small number of nodes to be in charge of the blockchain. In our system, all nodes can full-functionally participate in the consensus system, with their power proportional to their stake.

Blockchain is neutral regarding regulation.

—Guohua Gan

**Gan:** New trends also include the development of chain network technologies and cross-chain technologies, as well as the combination of blockchain with IoT and big data. In many cases, blockchain and IoT can share their nodes. IoT produces big data and blockchain offers a tool for data storage and trace.

**Guo:** What are the problems that need to be further studied?

**Zheng:** Chen mentioned the trilemma of blockchain. In my opinion, these three points—security, performance and decentralization—are the key indices of blockchain. So how to coordinate the optimization of these three indices and create blockchains suitable for certain scenarios will be a continuing topic.

**Gan:** That is right. Additionally, there are problems of capacity and communication. Limited by the block size, the amount of data that can be stored on the chain is currently limited. We have to balance the block size with performance. And to broadcast transactions across the chain, we need better support provided by communication technologies.

Additionally, as far as I know, the current blockchain, particularly the smart contract, has not been mathematically described and proved to be rational.

**Deng:** It is difficult to have a general algorithm to determine whether such a system is rational, as many blockchains, including ETH, are actually Turing complete. In fact, I suppose that whether a blockchain needs to be Turing complete is a question worth discussing.

I think there is another issue that needs to be studied. Blockchain can be applied to many economic applications, and thus, some new economic problems will arise. These may be of great value to the future economy, where algorithms and protocols play bigger and bigger roles, and the labor force is joined by more and more robots.

**Chen:** There are similarities and differences between traditional economics and blockchain economics. For example, virtualized digital property on the chain is different from traditional property. Tokens on blockchain may be connected with a user's decision-making right and voting right. So the transaction of a token is not simply the transaction of the usage right, but should also include the transaction of the decision-making right and the voting right. It will be interesting to look into these similarities and differences between traditional and new-born economic modes.

## THE BALANCE BETWEEN DECENTRALIZATION AND REGULATION

**Guo:** Another important issue is the regulation of blockchain. Given its anonymous and decentralized nature, how can blockchain systems be supervised or regulated?

**Zheng:** Around the world, no technology can be applied without regulation. Technically speaking, blockchain and regulation are not incompatible. In fact, as a tamper-resistant and transparent data chain, blockchain may be easy to control. We need to develop a regulation-in-technology mode—we can add regulation nodes into the blockchain and thus obtain comprehensive and real-time monitoring data.

**Wang:** Blockchain accounts seem to be anonymous. However, it is possible for cryptanalysts to determine the relevance between blockchain accounts and their owners in the real world with the help of cryptographic techniques. Because cryptography, say hash functions and digital signatures, is the key of blockchain technology, cryptographic techniques, including cryptanalysis techniques, provide powerful tools for blockchain regulation.

**Gan:** That is right. Blockchain is neutral regarding regulation. It is anonymous and privacy-protected on the one hand, and transparent and tamper-resistant on the other hand. So I suppose there are two specific measures required to regulate blockchain. The first is a relative real-name system, and this may rely on the digital signature technology mentioned by Professor Wang. The second measure is what Professor Zheng proposed: the addition of regulation nodes.

In fact, I think regulation itself is one of the major applications of blockchain technology. Provided we can accurately record and trace who did what to which subject at which time and in which place with the technology of blockchain, we can achieve better traceability and regulation of many fields.

**Deng:** I have taken part in a discussion on the regulation of another technology and proposed a suggestion. I think this suggestion is also applicable to blockchain: companies should proactively advise the government and tell the government how to supervise this field. Advice from companies can help to create an interactive atmosphere between the government and companies, which is very helpful for the formation of a healthy regulatory system.

## HEADING FOR THE FUTURE

**Guo:** Standardization is important for the internet industry and communication industry. Are there any blockchain standards being discussed?

**Gan:** Yes. In China, I have participated in the establishment of the ‘Trusted Blockchain Standard’. This standard is led by the China Academy of Information and Communications Technology of the Ministry of Industry and Information Technology (MIIT), and has become a part of the standard system of the International Telecommunication Union (ITU). Additionally,

Security, scalability and decentralization are key factors in blockchain standardization.

—Jing Chen

the China Electronics Standardization Institute of MIIT is also actively participating in the related International Organization for Standards (ISO) standards.

**Wang:** The first step of the standardization of blockchain should be the standardization of its three key cryptographic algorithms: encryption algorithms, digital signatures and hash functions. Mature standards for encryption algorithms already exist. For hash functions, after MD5 (Message-Digest Algorithm 5) and SHA-1 (Security Hash Algorithm-1) of the NIST (National Institute of Standards and Technology, US Department of Commerce) were successfully attacked in 2004 and 2005, China and the United States both proposed new hash function algorithms, including SM3 (China's commercial cryptography algorithm 3) and SHA-3. Applications for ISO standards for these new algorithms have been submitted, and the standards will come out soon.

There should also be standards for the protocol framework of blockchain, which belongs to the scope of the cryptographic protocol layer. Multiple blockchain algorithms have come out in recent years, so I think it is not easy to obtain a protocol framework standard in a short time. However, we may have a unified standard in 5 or 10 years’ time, which will be suitable for different scenarios.

**Chen:** Establishing blockchain standards is becoming more and more pressing. Every day we see new blockchain systems claiming different performance and security guarantees. End users have no way of looking into the techniques to understand the underlying trade-offs. Academia, industry and government need to be the driving forces for standardization.

It's important to choose common performance metrics, build analysis vectors and develop mathematically solid guarantees. Security, scalability and decentralization are key factors in blockchain standardization. For example, there are several degrees of participation mode: whether each node can directly and freely participate, whether a node has to delegate her participation right to others, and whether a node has to ‘lock-up’ her stake to participate. There are also several organization modes of the system: permissioned, consortium, permissionless, etc. Another factor in standardization is the underlying network structure: what is the required bandwidth, how are messages delivered, etc.

There can be many hidden factors in the system that affect its performance and are not communicated to end users. A lot of effort is needed in blockchain standardization to make the implicit explicit.

**Guo:** What is the current development stage of blockchain technology?

**Gan:** I think the development of blockchain technology roughly agrees with the hype cycle curve proposed by Gartner, Inc. Now, the development has crossed over the peak of inflated expectations and reached the trough. In China, the government is strict with virtual currency. And for blockchains without tokens, we are trying to promote applications in diverse fields. I think this period of technology accumulation and industrial adjustment will last for about 2 years, and we will see multiple real-world blockchain applications around 2020.

Our academic research should keep a certain distance from industry.

—Xiaotie Deng

**Figure 1:**
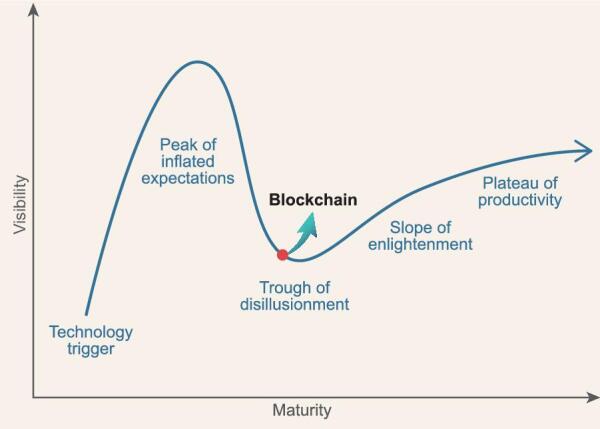
According to the hype cycle curve, the development of blockchain has crossed over the peak of inflated expectations and reached the trough.

**Deng:** I have considered a question about the development of blockchain for a long time: how many chains will survive in the end? Not long ago, Bitcoin Gold (BTG) was maliciously attacked, which indicates that the inter-attack among blockchains has begun. When the embryonic form of paper currency (交子, Jiaozi) first appeared in China, it was co-issued by 16 wealthy merchants. But in the end, the government became the only issuer. Now, EOS authorizes its token issuing power to 21 nodes. Will there be only one chain and one token remaining in the future or a list of them with sizes that follow the power law?

**Wang:** I think this question should be discussed as two separate cases. The first is the techniques relating to blockchain. For many scenarios that only need certain functionalities of blockchain, we can borrow certain techniques from blockchain to meet their demands. There are many types of such applications and the corresponding blockchain solutions can be diverse. But for the development of the entire blockchain framework, proposing a unified standard is an urgent task.

**Guo:** What are the similarities and differences between the research and development of blockchain in China and abroad?

**Gan:** I recently participated in a conference on the intellectual property of blockchain technology and learned that international blockchain patent applications are dominated by China and the United States. The numbers of applications for patents, both technical patents and application patents, of these two countries are similar. However, according to my personal feeling, even though we are very active and have invested a lot in the applications of blockchain, our basic research still lags. Moreover, foreign blockchains are generally combined with tokens, whereas we are mostly focused on blockchains without tokens.

**Deng:** Yes, there is a gap in basic research of blockchain between China and foreign countries. But there has been progress recently; more Chinese research groups are beginning to devote time to basic research on blockchain technology. In the United States, several Chinese scientists, including Jing Chen, Elaine Shi and Dawn Song, are making remarkable contributions to this field.

I think our academic research should keep a certain distance from industry. Basic research should be innovative and forward-looking. If researchers study only the technologies that have already been applied in industry, they will not be able to create new algorithms for the future. Now, many governments have taken quite a cautious attitude toward blockchain virtual currencies, which is important for practical financial purposes. But academic research may not want to drop out of this field full of challenges. Even though current technology is not completely mature yet, there is still a chance that such virtual currencies may play a significant role in international finance eventually. We may want to avoid the risk of missing the opportunity to have a fair share in the innovative international financial system forever, and at least be well positioned so that it does not cost much more to get back in.

## Notes

Weijie Zhao is a NSR news editor based in Beijing.

